# Urothelial carcinoma in situ of the entire urinary tract with invasion into seminal vesicle: A case report

**DOI:** 10.1016/j.eucr.2025.103185

**Published:** 2025-08-31

**Authors:** Vivien Wei-Anne Hsu, Nicole Swarbrick, Hemant Kulkarni, Andrew Redfern, Cynthia Hawks, Alarick Picardo, Steve P. McCombie, Dickon Hayne

**Affiliations:** a Department of Urology, Fiona Stanley Hospital, Perth, Western Australia, Australia; b UWA Medical School, University of Western Australia, Crawley, Western Australia, Australia; c PathWest Pathology & Laboratory Medicine, Perth, Western Australia, Australia; d Department of Nephrology, Fiona Stanley Hospital, Perth, Western Australia, Australia; e Department of Medical Oncology, Fiona Stanley Hospital, Perth, Western Australia, Australia

**Keywords:** Urothelial cancer, Carcinoma in situ, Seminal vesicle, Complete urinary tract extirpation, Renal transplant, Immunosuppression

## Abstract

Urothelial carcinoma in situ represents a high-grade, high-risk form of urothelial cancer which predominantly occurs within the bladder, rarely affecting other structures within the genitourinary system such as the seminal vesicle. Involvement could represent more advanced disease, necessitating more aggressive management. We present a case of CIS involving the entire urinary tract in a renal transplant patient who underwent a complete urinary tract extirpation (CUTE) for BCG-refractory CIS of the bladder. This case is unique, representing the first reported CUTE with malignant involvement of every site in the urinary tract.

## Abbreviations used:

BCG -: bacille Calmette-Guerin
BKV –: BK virus
CIS –: carcinoma in situ
CTIVU –: computed tomography intravenous urogram
CUTE –: complete urinary tract extirpation
FDG-PET –: fluorodeoxyglucose position emission tomography
MIBC –: muscle-invasive bladder cancer
PCKD –: polycystic kidney disease
PCR –: polymerase chain reaction
PLND –: pelvic lymph node dissection
TURBT –: transurethral resection of bladder tumour
SV –: seminal vesicle

## 1 Introduction

Urothelial carcinoma in situ (CIS) is a flat tumour confined to the bladder mucosa and not invading into subepithelial connective tissue, but is regarded as a high-grade, aggressive form of bladder cancer due to its high risk of progression,^1^ and should be managed equally aggressively. CIS predominantly affects the bladder with occasional occurrence in the upper tract, but does not commonly involve other structures of the genitourinary system. Involvement of the seminal vesicles (SV) by urothelial carcinoma is uncommon, with some literature reporting an incidence of 3 % in radical cystectomy cases.^2^ Involvement of seminal vesicles by CIS is even more rare, and may represent more advanced disease. According to the current TNM staging system, invasion of SV by urothelial carcinoma is classified as pT4, however the prognostic significance of mucosal spread of CIS to SV is uncertain^2^. Due to its rarity, the clinical significance remains unclear, which presents unique diagnostic and therapeutic challenges.

Current management for bladder CIS with bacille Calmette-Guerin (BCG) therapy is well-established.^3^ However, the role of BCG in treating disseminated or extravesical CIS is less clear and the extension of CIS to adjacent structures such as the seminal vesicles perhaps necessitates a more extensive surgical approach and possibly adjuvant therapies. The rarity of such cases limits the evidence base for optimal management.

We present a unique case of urothelial CIS involving every site in the urinary system including invasion into the SV in a renal transplant patient in the context of complete urinary tract extirpation (CUTE) for BCG-refractory CIS of the bladder.

## 2 Case

A 73-year-old man with BCG-refractory CIS of the bladder, on a background of necessary immunosuppression for a renal transplant for polycystic kidney disease (PCKD) and BK viraemia detected over 10 years ago, was referred for consideration of more radical surgical management. The patient had a history of recurrent urothelial carcinoma of the bladder with CIS detected on several occasions over the previous two years and T1 bladder disease identified on one occasion, despite two cycles of induction BCG and 8 instillations of maintenance BCG therapy. The pathology from his most recent procedure prior to referral had identified residual prostatic urethral disease, with evidence of intraductal and possibly stromal involvement. A recent CTIVU and FDG PET scan did not demonstrate any evidence of upper tract lesions (although the collecting systems of his non-functioning, polycystic, native kidneys were not well opacified), nodal or distant disease, but did demonstrate significant FDG-avidity in his prostate. His lower urinary tract symptoms were severe with marked urgency, frequency, bladder pain, dysuria and nocturia (of up to six times each night).

**Fig. 1:**
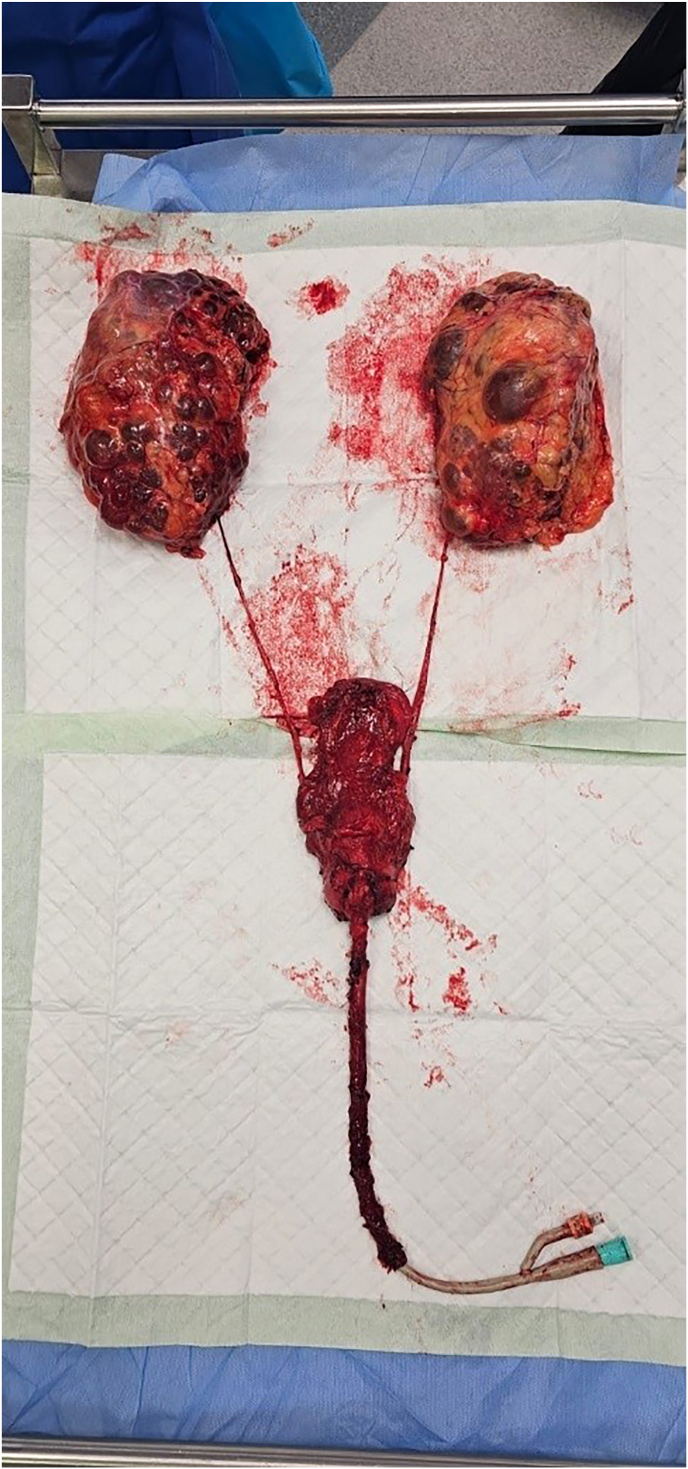
En bloc resection of native urinary tract including prostate and urethra.

Following consultation with his Nephrologist, discussion in a Uro-Oncology MDT and appropriate counselling, the patient underwent CUTE with laparoscopic bilateral nephroureterectomy and synchronous open cystoprostatourethrectomy with left pelvic lymph node dissection and formation of ileal conduit, with anastomosis of this onto his transplant ureter (Fig. 1). The surgical strategy in this case required careful consideration given the patient's renal transplant and the need for complete urinary tract extirpation. A laparoscopic approach to the bilateral nephroureterectomy was chosen to minimise morbidity given the patient's age and comorbidities. However, we proceeded with an open approach for the cystoprostatourethrectomy due to anticipated technical complexity, including pelvic adhesions, and the need for safe dissection and mobilisation around the transplant ureter and vascular pedicle. The donor kidney, located in the right iliac fossa, constrained the operative field and influenced our decision to omit a right-sided pelvic lymph node dissection due to the high risk of compromising the graft vessels. Additionally, the urinary diversion required anastomosis of the ileal conduit directly to the transplant ureter. This step demanded meticulous handling to ensure a tension-free anastomosis and unobstructed drainage while avoiding ureteric devascularisation. These intraoperative decisions highlight the unique challenges of performing genitourinary extirpative surgery in transplant recipients.

**Fig. 2:**
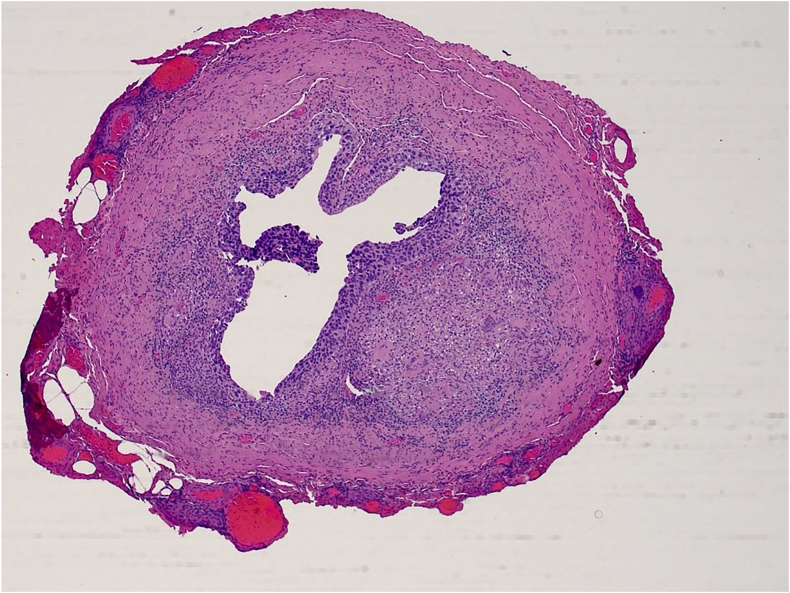
Transverse section of right ureter, showing extensive urothelial CIS as well as a granuloma within the underlying stroma (HE, ×12.5 magnification).

**Fig. 3:**
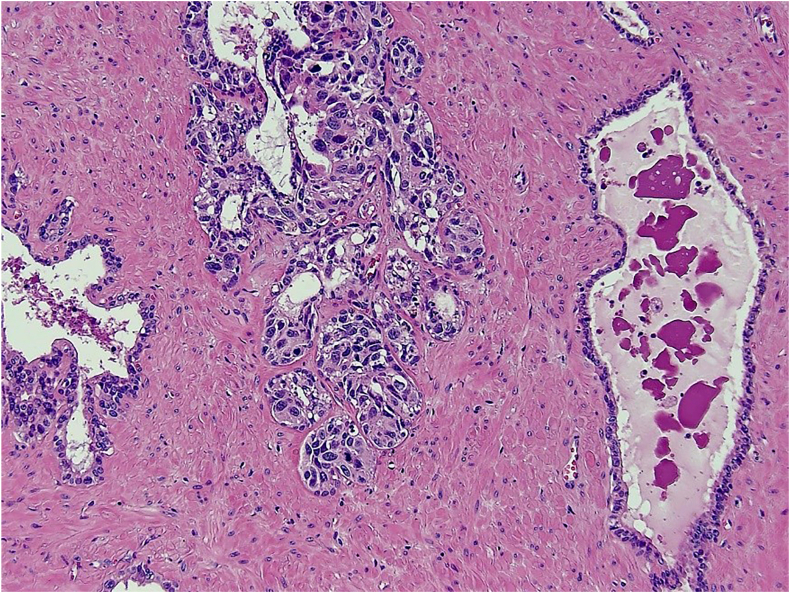
Comparison between normal SV mucosa (right) and SV mucosa colonised by urothelial CIS (centre/left – as indicated by arrow) (HE, ×100 magnification).

**Fig. 4:**
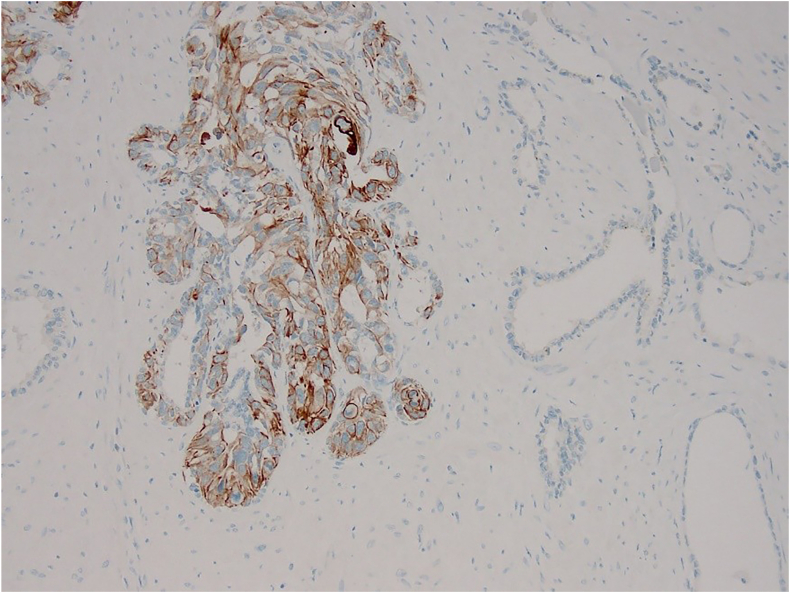
Immunohistochemistry for Cytokeratin 20 highlights the urothelial CIS cells, leaving the normal SV mucosa unstained (CK20 IHC, ×100 magnification).

Cystoscopy on the day of surgery showed papillary changes to the prostatic urethra without any obvious tumours within the bladder. The decision was made not to perform a pelvic lymph node dissection on the right due to anticipated risk to the donor kidney in the right iliac fossa. Subsequent histopathology (Fig. 2, Fig. 3, Fig. 4) following CUTE showed pT1 disease in the right renal pelvis and extensive CIS involving both native pelvicalyceal systems, bilateral ureters, bladder, prostatic urethra, expanding prostatic ducts and extending into the left seminal vesicle, as well as involving the proximal penile urethra. The patient's postoperative course was uncomplicated, he was discharged on post-operative day 9, and he remains well at the time of publication on regular surveillance (Table 1).

**Table 1:** Summary table showing overview of patient demographics, histopathology and current status.

Patient	BMI	Surgery	Site of malignancy	Preoperative histopathology diagnosis	Postoperative histopathology	Reason for renal transplant	BKV- induced	Neoadjuvant/Adjuvant Therapy	Current status (as of June 2024)
73M	25	Bilateral nephroureterectomy + cystoprostatourethrectomy + left PLND + ileal conduit (June 2024)	Bladder and prostate – urothelial carcinoma with significant intraductal component and possible involvement of prostatic stroma	CIS	CIS involving both pelvicalyceal systems, ureters, bladder, prostatic urethra, expanding prostatic ducts and extending into left seminal vesicle and proximal penile urethra + Gleason 3 + 3 = 6 prostate adenocarcinoma; limited to prostate, clear of margins	ESRF secondary to PCKD (2006)	N	N – previous chemotherapy for lymphoma	Alive – functioning transplant

## 3 Discussion

Management of CIS of the bladder is challenging due to the difficulty in identification of the disease, and its aggressive nature, with it being associated with a high rate of recurrence progression. Intravesical instillation of BCG is the established first-line treatment for CIS, with radical cystectomy generally reserved for BCG-refractory disease,^4^ and bladder-sparing options such as intravesical chemotherapy reserved for patients unsuitable for cystectomy. However, this case highlights unique diagnostic and therapeutic challenges with extravesical CIS, the prognostic significance of which is uncertain, and with the patient's state of lifelong immunosuppression following renal transplantation. Theoretically, immunosuppression is a contraindication to immunotherapy such as BCG due to the risk of sepsis and morbidity.^5^ BCG induces an endovesical inflammatory reaction and increases cytotoxic agents to act directly on tumour cells. This presents a contradictory dilemma for transplant patients as immunosuppression is required to avoid renal transplant rejection, but immunologic activity is necessary for a cytotoxic effect on tumour cells, and thus renders BCG less effective in the immunosuppressed transplant patient. Limited literature exists on the use of BCG to treat bladder cancers in post-transplantation patients and it remains unclear whether immunosuppression should be regarded as an absolute contraindication to immunotherapy.

SV involvement by CIS is rare and may indicate an advanced stage of disease or direct extension from the bladder. Two distinct patterns have been suggested in the literature to explain how primary urothelial carcinoma of the bladder extends to the SV; direct invasion through the bladder wall and perivesical fat, or pagetoid mucosal spread.^6^ Direct invasion is the predominant mechanism, while pagetoid spread is uncommon. Irrespective of the mechanism of extension outside of the bladder, involvement of SV by CIS signifies more aggressive and advanced disease and may be associated with a worse prognosis compared to CIS confined to the bladder.

Additionally, this case presents further unique features with the patient being a renal transplant patient with BK-viraemia detected. However, despite BKV being previously detected in urine, the CUTE specimen was found to be BKV DNA negative and thus not confirmed to be BKV-induced malignant disease. This case poses an interesting point on the potential role of BKV in malignancies of the urinary system. BK virus (BKV) is a polyoma virus which remains latent within the urinary tract of healthy immunocompetent individuals after initial infection and can reactivate in immunosuppressed patients to re-establish infection, with a risk for development of urothelial carcinoma.^1^ The exact pathogenesis is unknown; however, limited literature exists which describes an association between BKV and urothelial carcinoma of the bladder in the setting of post-renal transplantation immunosuppression.^7^ Malignancies in transplant patients have been theorised to be more aggressive, and the state of immunosuppression in renal transplant patients makes BKV-induced urothelial cancers challenging to manage.^7^ Given that BKV-induced urothelial cancer is considered to be an aggressive disease and the increased risk of malignancy associated with lifelong immunosuppression in transplant patients, radical cystectomy with excision of the native urinary tract is not an unreasonable consideration for muscle-invasive bladder cancer and recurrent CIS in renal transplant patients.

## 4 Conclusion

Urothelial CIS involving the entire urinary system with invasion into the SV is an extremely rare but significant clinical entity and represents a challenging clinical scenario. This case underscores the importance of considering more aggressive management in patients with CIS who do not respond to standard treatment modalities and demonstrates the need for heightened awareness and comprehensive diagnostic evaluation in patients with atypical involvement of CIS. Further research is warranted to better understand the natural history and optimal treatment strategies for such advanced manifestations.

## CRediT authorship contribution statement

**Vivien Wei-Anne Hsu:** Writing – review & editing, Writing – original draft. **Nicole Swarbrick:** Writing – review & editing, Formal analysis. **Hemant Kulkarni:** Writing – review & editing. **Andrew Redfern:** Writing – review & editing. **Cynthia Hawks:** Methodology, Data curation. **Alarick Picardo:** Writing – review & editing. **Steve P. McCombie:** Writing – review & editing, Conceptualization. **Dickon Hayne:** Writing – review & editing, Supervision, Conceptualization.
